# Medical Department of the Army

**Published:** 1865-10

**Authors:** 


Medical Department of the Army.—Maj. General Sickles paid the following compliment in a public address to the medical department of the army:

"I can do no more than glance at the improvement of the medical department of the army; indeed I can only speak of any of the great staff department so far as their operations passed under my own observation. The construction and organization of general hospitals, the ample arrangement for field hospitals, the liberal and various supplies for hospitals, the unstinted and judicious expenditures for scientific appliances, improved ambulances, hospital wagons—which are portable apothecary shops—

hospital cars, adapted expressly with spring-beds to carry the sick and wounded of the army over railroads; the humane use of chloroform, the liberal supply of stimulants; the extensive issue of quinine, one of the most expensive medicines, as a preventive—these are among the noticeable features of our improved administration of the medical service in the army."

